# Projecting cancer prevalence by phase of care: a methodological approach for health service planning

**DOI:** 10.3389/fonc.2023.1201464

**Published:** 2023-08-30

**Authors:** Silvia Francisci, Francesco Tursini, Luigino Dal Maso, Anna Gigli, Stefano Guzzinati

**Affiliations:** ^1^ National Center for Disease Prevention and Health Promotion, National Health Institute, Rome, Italy; ^2^ Institute for Research on Population and Social Policies, National Research Council, Rome, Italy; ^3^ Cancer Epidemiology Unit, Centro di Riferimento Oncologico di Aviano (CRO) Istituto di Ricovero e Cura a Carattere Scientifico (IRCCS), Aviano, Italy; ^4^ Regional Epidemiological Service, Veneto Cancer Registry (RTV), Azienda Zero, Padova, Italy

**Keywords:** cancer prevalence, phase of care, cancer registry, projections, survivorship, health service planning

## Abstract

**Background:**

In most developed countries, the number of cancer survivors is expected to increase in the coming decades because of rising incidence and survival rates and an aging population. These patients are heterogeneous in terms of health service demands: from recently diagnosed patients requiring first-course therapy to patients with extensive care needs and severe disabilities to long-term survivors who only need minimal care. Therefore, in terms of providing healthcare planners and policymakers with useful indicators for addressing policies according to health service demands, it is worth supplying updated measures of prevalence for groups of patients based on the level of care they require. The aim of this paper is to illustrate a new method for estimating short-term projections of cancer prevalence by phase of care that applies to areas covered by cancer registration.

**Methods:**

The proposed method combines linear regression models to project limited duration prevalence derived from cancer registry data and a session of the freely available software COMPREV to estimate the projected complete prevalence into three distinct clinically relevant phases of care: initial, continuing, and final. The method is illustrated and validated using data from the Veneto region in Italy for breast, colorectal, and lung cancers.

**Results:**

Prevalence is expected to increase in 2015-2026 for all considered cancer sites and sexes, with average annual variations spanning from 2.6% for women with lung cancer to 0.5% for men with colorectal cancer. The only exception is lung cancer prevalence in men, which shows an average annual decrease of 1.9%. The majority of patients are in the continuing phase of care, followed by the initial and final phases, except for lung cancer, where the final phase of care prevails over the initial one.

**Discussion:**

The paper proposes a method for estimating (short-term) future cancer healthcare needs that is based on user-friendly and freely available software and linear regression models. Validation results confirm the applicability of our method to the most frequent cancer types, provided that cancer registry data with at least 15 years of registration are available. Evidence from this method is addressed to policymakers for planning future cancer care, thus improving the cancer survivorship experience for patients and caregivers.

## 1 Introduction

In most European countries, prevalent cases make up an important share of the whole population; according to recent estimates, prevalent cases of cancer in Italy accounted for 5.7% of the national population in 2020 (1), corresponding to 3.6 million inhabitants. These absolute numbers are forecast based on an increasing trend of 3.2% per year in the first decade of the 2000s, which is consistent with estimates reported for the USA (2.8% per year) (2), Switzerland (3), and the UK (4). Cancer prevalence is a function of incidence and survival. It increases when new cases are diagnosed and decreases when cancer patients die. Moreover, population growth and changes in the age structure of the population have a relevant impact on prevalence since the risk of cancer increases with age (5).

Cancer survivors represent a growing population because of increases in cancer survival, due to advances in treatment and early diagnosis, and the aging of the population, and the impact of these trends is exceeding the declining incidence observed for some neoplasms (6, 7). Cancer survivors have complex health problems and are heterogeneous in their needs for medical care, psychosocial support, and practical assistance (1, 3). They are generally classified according to the length of survival time and disease outcome, and the vast majority of cancer survivors diagnosed with the most common cancer types survive more than 5 years after diagnosis (8). Most of them receive cancer-related medical care at diagnosis, and some will receive cancer care throughout the rest of their lives. Therefore, medical care expenditures associated with cancer are substantial and are projected to increase dramatically in the near future (9).

Cancer prevalence represents a fundamental measure of cancer burden and cancer survivorship (10). It includes all survivors, irrespective of their patterns of care, and is therefore not suitable to inform healthcare planning, resource allocation, or cost estimation. To overcome this limitation and to better understand the burden of cancer on the healthcare system, several studies have proposed and implemented a breakdown of prevalence into phases of care, i.e., clinically relevant periods related to diagnosis and death (11–13). Different stakeholders are interested in estimating and forecasting cancer prevalence by phase of care: policymakers, to plan sustainable healthcare policies and resource allocation according to the needs of cancer survivors; epidemiologists, to describe the impact of cancer in the population, taking into account the combined effect of incidence, survival, and demographic changes; clinicians, to develop guidelines to improve standardized medium- and long-term follow-up of cancer survivors; and patients, to find support for a complete social recovery and to better meet their rehabilitation needs (1, 13).

Estimates of prevalence are commonly based on limited duration prevalence (LDP) derived from population-based cancer registry data. However, LDP only includes cancer survivors who were diagnosed during the period of activity of the cancer registry, and the shorter this period, the lower the LDP measure (14). Moreover, data collection is retrospective, and the delay between the present time and the time of registration is at least three years (15). To overcome these drawbacks, there are well-consolidated statistical models to estimate complete prevalence, which includes all persons diagnosed with cancer in a given population who are alive at a given prevalence date, regardless of how long ago they were diagnosed (16, 17). However, it is necessary to have more updated prevalence figures than those derived from cancer registry data and to be able to break down complete prevalence by phase of care to account for the heterogeneity of cancer survivors with respect to their healthcare needs.

These needs are addressed in this study, which aims at presenting a methodological approach to project the complete prevalent population by phase of care in the near future. This approach combines methods specifically developed for deriving LDP from population-based cancer registries, using the counting method implemented in the SEER*Stat software (18); implementing short-term projections of LDP; estimating projected complete prevalence in three distinct clinically relevant phases of care - the initial phase following diagnosis, the last year of life, and the continuing phase in between - using the completeness index method - as implemented in the COMPREV software (19).

The method is illustrated and validated using cancer registry data from the Veneto region (Italy), which have been collected in the framework of the Epicost-2 study (20). The method was applied to forecast prevalence by phase of care in 2025 for the following cancer sites: breast (female subjects), colon and rectum (male and female subjects), and lung (male and female subjects).

## 2 Materials and methods

### 2.1 Definitions

Limited-duration prevalence (LDP) is the number of people who are alive on a certain date X and have had a cancer diagnosis in a limited period. The maximum duration of this period depends on the number of years the registry has been collecting incidence cases. LDP is calculated from cancer registry data using the SEER*Stat software (18). When running the limited-duration session in SEER*Stat, the option “All Tumors Matching Selection Criteria/One Tumor Per Statistic” was used. According to that option, LDP refers to person prevalence: that is, a person will not contribute to a single prevalence estimate with more than one tumor diagnosis.

Complete prevalence (CP) is the number of people who are alive on a given date X with a prior diagnosis of cancer, regardless of when the diagnosis occurred. CP is estimated using LDP and the completeness index method to estimate survivors diagnosed before cancer registration (16).

P is the proportion of LDP per 100,000, i.e., the ratio of the number of cases in a specific population to the population itself.

### 2.2 Data sources

We used data from the Veneto Cancer Registry (VCR), a population-based cancer registry that covers approximately 2.1 million inhabitants (43% of the whole region) in northeastern Italy. Patients diagnosed with colon and rectum, lung, and breast (female subjects only) cancer between 1990 and 2018 were selected and followed up for vital status until 31/12/2019.

### 2.3 Input data

LDP and P matrices were stratified by single year of age at prevalence date (t= 0,…, 84, 85+) and by single-year duration d, intended as the distance in years from diagnosis to prevalence date. LDP and P matrices are derived for the more recent five years of incidence: from 01/01/2015 to 01/01/2019. These matrices are the input data for projections; each LDP corresponds to a different maximum duration, and the maximum common duration is 25 years.

Completeness indices were obtained from parameter estimation of survival and incidence models from eight historical Italian cancer registries in the period 1985-2009 (1).

### 2.4 Projecting limited duration prevalence

We assumed that the prevalence proportion P follows a linear trend in time based on the trend of the last five calendar years. The assumption of a linear trend in P is reasonable for short- or medium-term (e.g., 10-year) projections (1). The steps below were applied to the five LDP matrices from 01/01/2015 to 01/01/2019 to derive the CP by phase of care projected to 01/01/2025 in the population covered by the VCR.

The projection algorithm is made up of the following steps:

i. Compute the LDP proportion (P) (summed for all ages and durations) in the last 5 years of observation (from 01/01/2015 to 01/01/2019 in our example):


(1) $$P\left(x\right)=\frac{\sum_{t=1}^{T}\sum_{d=1}^{D}LDP\left(t,d,x\right)}{\sum_{t=1}^{T}Pop\left(t,x\right)}\times 100,000$$

where LDP (t,d,x) is the number of prevalent cases of age t and duration d alive on prevalence date x (=2015,…, 2019), Pop(t,x) is the population of the area covered by the VCR on prevalence date x, stratified by age t, maximum age is T=85+ years, and the maximum common duration is D=25 (incidence data from 1990 to 2018).

ii. Fit a linear regression to the LDP proportion for all ages and durations combined


(2) P(x)=α+βx

where the dependent variable is the prevalence proportion P and the covariate is the prevalence date x (=01/01/2015,…, 01/01/2019) and obtain the estimates of the two parameters: $\hat{\alpha }$ and $\hat{\beta }$ .

iii. Project the linear regression in year X (in our example, 01/01/2025) to obtain the projected prevalence proportion


(3) $$\hat{P}\left(X\right)=\hat{\alpha }+\hat{\beta }\times X$$

The 95% prediction intervals of the projected prevalence proportion were calculated using the “predict” function in R software (21).

iv. Compute the distribution of prevalent cases in the last available year (01/01/2019 in our example). For each age t and duration d we have:


(4) $$w\left(t,d,2019\right)=\frac{LDP\left(t,d,2019\right)}{\sum_{t=1}^{T}\sum_{d=1}^{D}LDP\left(t,d,2019\right)}$$

Where LDP (t, d, 01/01/2019) is the number of cases of age t and duration d alive on 01/01/2019

v. For each annual age t and annual duration d, compute the projected prevalent cases in year X:


(5) $$\hat{LDP}\left(t,d,X\right)=\hat{P}\left(X\right)\times Pop\left(X\right)\times w\left(t,d,2019\right)$$

where Pop(X) is the projected population of the region (computed by the Italian National Institute of Statistics ISTAT (22)) in year X, and w(t,d,2019) are the weights computed in (4) reflecting the distribution of prevalent cases by age and duration in the latest prevalence date of available observations (01/01/2019).

vi. Repeat steps iii and v for year X+1 (in our example, 01/01/2026); notice that the same weights computed in iv are used for the calculation of projected LDP in year X+1.

The projected LDP matrices $\hat{LDP}$ (t, d, X) and $\hat{LDP}$ (t, d, X+1) will be used to decompose the projected Complete Prevalence in year X by phase of care.

### 2.5 Decomposing the projected complete prevalence by phase of care

The COMPREV software (23) allows estimating the complete prevalence by phase of care, i.e., to break down the complete prevalence into three mutually exclusive phases: the initial phase (Ini, the first 12 months after diagnosis), the end-of-life phase (EOL, or final, i.e., 12 months before death), and the continuing phase (Cont), defined as the time in between initial and EOL. At the prevalence date, each patient belongs to one of these phases, according to the date of diagnosis and life status: a patient diagnosed within 12 months before the prevalence date and alive 12 months after the prevalence date belongs to the initial phase (Ini); a patient diagnosed more than 12 months before the prevalence date and alive 12 months after the prevalence date belongs to the continuing phase (Cont); and a patient who died within 12 months after the prevalence date, regardless of when they were diagnosed, belongs to the EOL phase (EOL). The EOL phase can be further subdivided into EOL cancer (prevalent cases whose death is due to cancer) and EOL other cause (prevalent cases whose death is due to causes other than cancer), according to the cause of death. This breakdown of the final phase is feasible when information on the cause of death is available.

COMPREV requires the input of two LDP data files: the first one refers to year X, and the second one must refer to the successive year X+1; these files must be identical in their settings except for the year of prevalence to which they refer and must be stratified by single ages at prevalence and single year durations (19). COMPREV also requires completeness indices, specific to cancer type and sex, obtained by statistical regression models of incidence and survival data from cancer registries. A survival matrix containing a crude probability of death is also required to break down the EOL phase into EOL cancer and EOL other causes.

We applied COMPREV to the LDP projected matrices (5), which were computed at prevalence dates X and X+1, to obtain an estimate of the projected complete prevalence by phase of care in year X, stratified by age at prevalence:

CP(t,X,Ini), CP(t,X,Cont),CP(t,X,EOL), where CP(t,X,Ini)+ CP(t,X,Cont)+CP(t,x,EOL)= CP(t,X).

### 2.6 Validation of the projected complete prevalence by phase of care

In order to validate the method, we applied the above-illustrated algorithm to a subset of the VCR data, comprising patients diagnosed with colon and rectum, lung, and breast (female subjects only) cancer in 1990-2011 and followed for vital status until 01/01/2012:

- derive LDP in five consecutive years, 2008-2012, with a maximum common duration of 18 years;
- project LDP proportions in the years 2018 and 2019, as described in Section 2.4;
- compute the projected complete prevalence by phase of care on 01/01/2018 via COMPREV, as described in Section 2.5;
- directly estimate the complete prevalence by phase of care by applying completeness indices to the LDP in years 2018 and 2019 derived from the complete set of VCR data (i.e., patients diagnosed over the entire period of data availability 1990-2018 and followed up to 01/01/2019);
- compare the projected and estimated complete prevalence by cancer site and phase of care.

The results of this validation are illustrated in **Supplementary Table 1** and **Supplementary Figure 1**.

We also investigated the minimum length of cancer registry data required for the projections by comparing the projected complete prevalence by phase of care in 2025 using 25-year LDP data (incidence data period 1990-2018, follow-up 01/01/2019) with that obtained using 15-year LDP data (incidence data period 2000-2018, follow-up 01/01/2019.

The results of this validation are illustrated in **Supplementary Figure 2**.

## 3 Results

**Figure 1:**
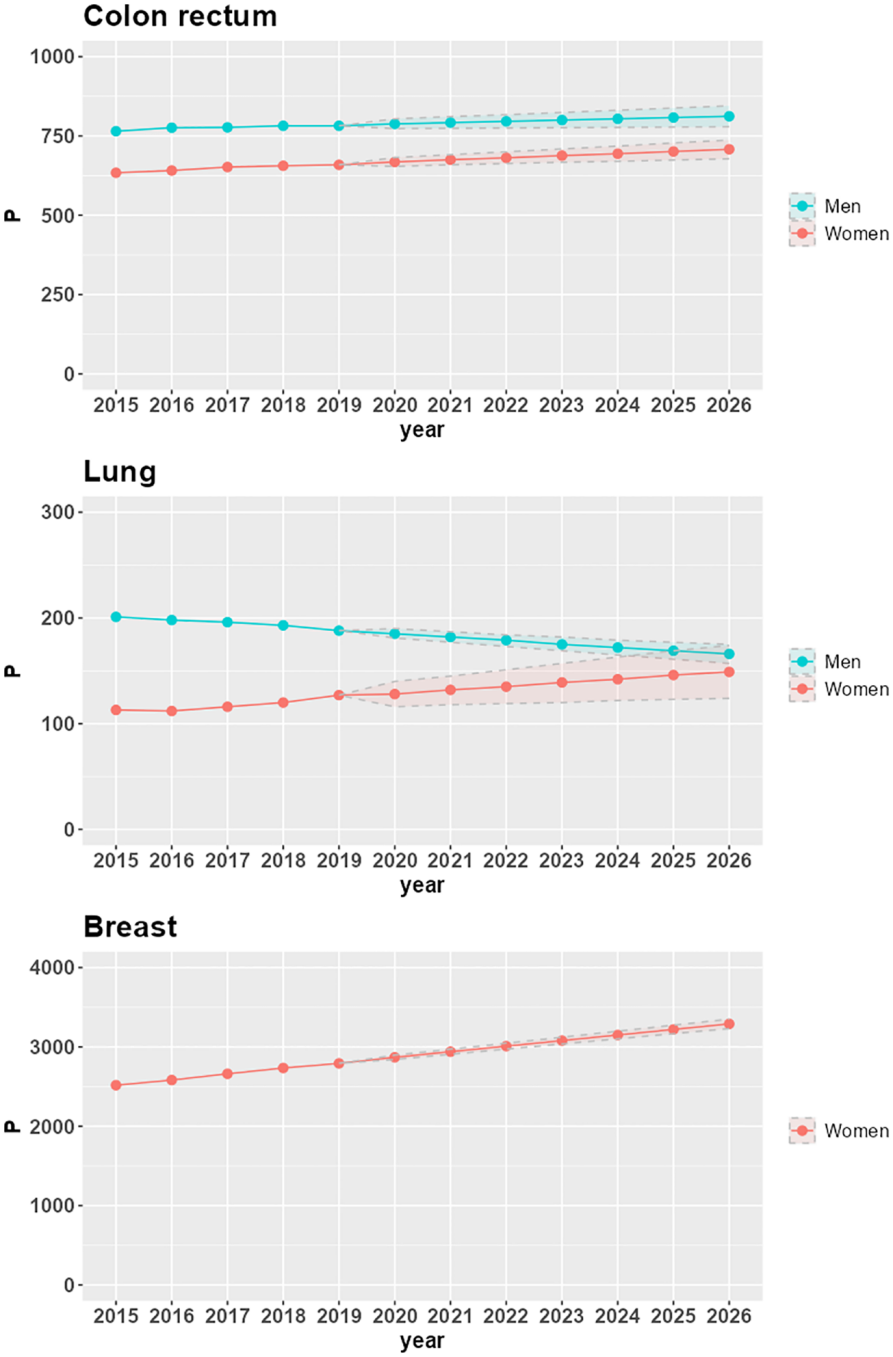
Time trends of 25-year LDP proportions per 100,000 (P), with lower and upper bounds of 95% confidence intervals, by cancer site and sex in the Veneto region.

**Figure 1** shows time trends of 25-year LDP proportions P by cancer site and sex in the Veneto region. From 2015 to 2019, the proportions are based on VCR data; from 2020 to 2026, the proportions are projected via linear regression; the lower and upper bounds of the projections are also included in the figure.

P increases in the seven-year projection period 2020-2026 for all combinations of cancer site and sex, except for lung cancer in the male population (-10.6%, corresponding to a -1.9% average annual variation). The largest increases are in women with lung cancer (16.5%, corresponding to a 2.6% average annual variation), breast cancer (14.7%, corresponding to a 2.3% average annual variation), and colorectal cancer (5.9%, corresponding to a 1% average annual variation). In men, there is a 3% increase in P for colorectal cancer (corresponding to a 0.5% average annual variation).

The increasing trends in LDP proportions derive from increasing incidence (as is the case for lung cancer in women) or stable incidence (as is the case for breast cancer) combined with population aging. For colorectal cancer, the reduction in the risk of developing the disease, which led to a decrease in incidence in 2007-2008 for both men and women (24), does not yet compensate for the combined effect of aging and increasing survival, thus resulting in a slight but positive trend until 2026.

The decreasing trend of LDP proportions in men diagnosed with lung cancer is due to a decrease in incidence: in the Veneto region, the APC (Annual Percent Change) incidence spans from -1.3% in the 1990s to -3.7% at the beginning of the 2000s (25). Despite the aging of the population and the increase in survival, this decrease in incidence determines the reduction of prevalence: from 201 prevalent cases per 100,000 in 2015 (corresponding to 4,809 patients overall in the Veneto region) to 166 prevalent cases per 100,000 in 2026 (corresponding to 3,927 patients overall in the Veneto region).

The projection of prevalent cases decomposed by phase of care is the main application of the methodology, and the results are illustrated in **Table 1**. The total number of prevalent cases (complete prevalence) estimated in 2018 and projected in 2025 in the Veneto region are reported by phase of care and age group at prevalence (<50, 50-69, 70+). During the 7 years, the complete prevalence shows an increase in the percent variation between 6% for colorectal cancer in men, where the number of survivors increases from 19,342 to 20,436, and 25% for lung cancer in women, where the number of survivors increases from 3,010 to 3,750. The only exception is lung cancer in men, showing a 12% decrease in the percent variation with the number of survivors decreasing from 4,848 to 4,263.

**Table 1:** Complete prevalence (counts) estimated in 2018 (CP 2018) and projected in 2025 (CP 2025) in the Veneto region by cancer site, age group at prevalence, and phase of care.

CP 2018		Phase of Care				CP 2025		Phase of Care			
Cancer Site - Sex	Age Group	Initial	Continuing	Final	Total	Cancer Site - Sex	Age Group	Initial	Continuing	Final	Total
Colon Rectum-M	<50	53	341	7	400	Colon Rectum-M	<50	53	347	6	407
	50-69	553	4753	251	5557		50-69	578	4972	222	5773
	70 +	752	11566	1067	13385		70 +	810	12435	1011	14257
	all ages	1358	16660	1324	19342		all ages	1441	17755	1239	20436
Colon Rectum-F	<50	69	366	19	453	Colon Rectum-F	<50	71	384	17	472
	50-69	390	4079	161	4630		50-69	415	4386	133	4935
	70 +	706	10882	690	12278		70 +	754	12073	753	13580
	all ages	1164	15327	869	17361		all ages	1241	16843	903	18987
Breast-F	<50	811	4647	104	5562	Breast-F	<50	928	5302	110	6341
	50-69	2182	27706	467	30355		50-69	2548	32592	493	35632
	70 +	1658	36312	1637	39606		70 +	2049	44078	2013	48139
	all ages	4651	68665	2208	75523		all ages	5525	81971	2616	90112
Lung-M	<50	17	58	18	93	Lung-M	<50	16	54	14	84
	50-69	206	806	257	1269		50-69	180	731	218	1129
	70 +	342	2306	839	3487		70 +	305	2011	733	3049
	all ages	565	3170	1114	4848		all ages	501	2796	965	4263
Lung-F	<50	21	76	15	112	Lung-F	<50	24	103	21	148
	50-69	184	720	168	1072		50-69	214	867	238	1319
	70 +	223	1378	224	1825		70 +	274	1633	377	2284
	all ages	429	2174	406	3010		all ages	513	2602	635	3750

For women with breast cancer and men with lung cancer, variations are evenly distributed by phase of care, with an increasing trend by age. For men with colorectal cancer, the complete prevalence increases between 6% and 7% in the initial and continuing phases of care, respectively, and decreases by about 6% in the final phase of care. For women, most of the variation is due to the increase in survivors in the continuing phase of care (10% percent variation, from 15327 to 16843 patients). For women with lung cancer, most of the variation is due to the increasing number of survivors in the final phase of care (from 406 to 635 women), thus representing an increasing share of the prevalence cohort (from 14% in 2018 to 17% in 2025).

Major variations concern the elderly population, aged 70 years and over. Time trends and patterns by age at prevalence are due to the aging of the population and the consequent increased risk of developing cancer.

In the initial phase of care, the increase in prevalence for colorectal cancer is less pronounced among patients aged 50 to 69, possibly as a consequence of screening programs that allow the detection of pre-cancerous lesions, thus reducing the number of newly diagnosed patients; in all phases of care, the increase in the number of lung cancer survivors among the female population is higher for women aged 15 to 49, consistent with the increasing prevalence of smoking among young women (26).

**Figure 2:**
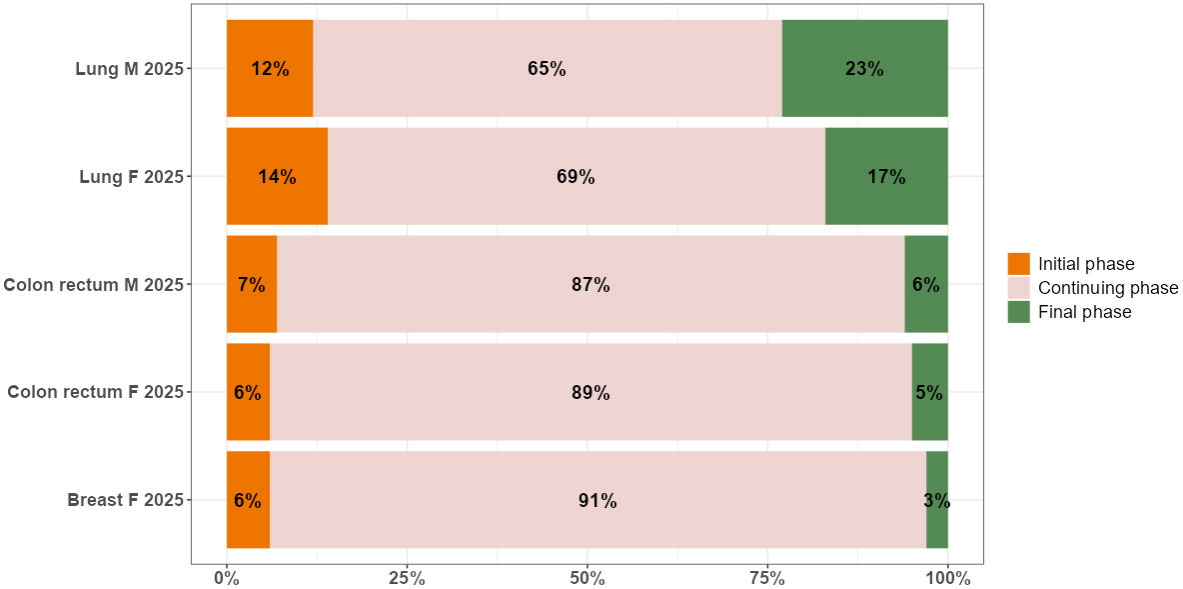
Distribution by phase of care (% values) of complete prevalence in 2025, by cancer site and sex in the Veneto region.

The bar plot in **Figure 2** presents the breakdown of complete prevalence by phase of care in 2025 by cancer site and sex.

The dynamics of prevalence during the projection period from 2018 to 2025 slightly affect the distribution of cancer survivors in the three phases of care: most patients are in the continuing phase of care, followed by the initial and final phases, except for lung cancer, where the percentage of patients in the final phase is higher than in the initial one. There are two patterns according to survival: among cancer patients with a better prognosis, as is the case for women with breast cancer, and all patients with colorectal cancer, 87%-91% are in the continuing phase, 6%-7% are in the initial phase, and 3%-6% are end-of-life patients. Among cancer patients with poorer prognoses, as is the case for all patients with lung cancer, 66%-69% are in the continuing phase, 12%-14% are in the initial phase, and 17-23% are end-of-life patients.

## 4 Discussion

Estimating and projecting cancer prevalence according to different phases of care is a prerequisite for estimating the economic impact of cancer.

The estimation of cancer prevalence by phase of care was mentioned in 2001 by Brown et al. in their seminal paper on the economic burden of cancer (27). Since then, many researchers have contributed to the field (11, 13, 28, 29). Estimation of cancer prevalence by phase of care is feasible when longitudinal data, identified at the level of individual-incidence cancer cases, are available. Cancer registries typically collect these data.

Prevalence is a complex indicator that depends on incidence, survival, and population dynamics. These determinants are to be taken into account in the projection of prevalence. A step forward in this direction was the projection of cancer prevalence based on a deterministic relationship between cancer mortality, incidence, and survival: the PIAMOD approach (5), which estimates and projects cancer prevalence as a function of incidence and survival models, with minor *ad hoc* hypotheses on the population evolution patterns. In PIAMOD, a linear period trend is assumed for incidence projections. For survival projections, two hypotheses are proposed: a conservative one, which assumes that cancer patient survival will remain stable for the projected years, and an optimistic one, which assumes that cancer patient survival will continue to improve at the same rate as observed in recent years (5).

PIAMOD was used as a basis for projecting prevalence by phase of care by Mariotto et al. (30) and later by Yu et al. (31). It is used for purposes similar to our method, but it requires more data (incidence and survival) and modeling than our approach. On the other hand, PIAMOD is more flexible as it allows one to distinguish the contribution of incidence from that determined by survival in the prevalence projections.

According to Yu, this approach has some drawbacks: *“The process involves many decisions to be made, such as selecting a high-dimensional polynomial incidence model and mixture cure model for relative survival based on different assumptions for future trends. All of these decisions must be informed by a high level of cancer epidemiological and statistical knowledge, and the resulting prevalence estimates are highly dependent on these modelling decisions and assumptions.”*

In this paper, we propose an alternative semi-parametric approach that combines the projection of LDP data from CR (1) and the decomposition of the projected prevalence into phases of care (19).

This approach is quite straightforward and does not require complex modeling, as completeness indices are externally estimated from other studies. It provides good results for the most frequent cancer types, which are the most interesting cases from the perspective of estimating the economic burden. To decompose the projected prevalence by phase of care, we used the software COMPREV, which is freely available and easy-to-use; the software contains a set of default parameter estimates obtained from SEER (Surveillance, Epidemiology, and End Results) data. Further, population forecasts can easily be embedded; for Italy, these were provided by the Italian Institute of Statistics (ISTAT). Finally, the method works equally well on shorter incidence data series (15 years); thus, it can also be applied to more recently established CRs and allows one to incorporate more recent trends in the projections, as has been shown in the case of colorectal cancer.

There are some limitations:

The method does not allow one to project prevalence according to different scenarios of incidence and survival dynamics. However, according to a sensitivity analysis presented in previous studies (31), approximately 71% of the prevalence dynamics reflect the impact of population growth and aging, while the remaining 29% are attributable to incidence and survival changes.

The phase of care decomposition does not function well when the number of cases is too small, especially when the scarce numbers are concentrated in only one of the phases of care, such as for thyroid cancer, where there are virtually no cases in the end-of-life phase. We must bear in mind, however, that less frequent cancers have a smaller economic impact. Therefore, the applicability of the proposed methodology is limited to the most frequent cancers that have a substantial economic impact on the healthcare system, and to a short- to medium-term forecast horizon, which is typically considered for planning healthcare intervention policies. Within this context, the proposed complete prevalence projections by phase of care have been validated and produce reliable results. The continuing phase includes patients who may be highly heterogeneous in terms of healthcare: some of them have recently completed their initial therapy and require follow-up, some others require treatment for cancer recurrence or second primary cancers and, finally, some have survived for a long period since their initial treatment and can be considered cured.

Further developments of this method can be considered:

Data on specific treatments and procedures collected in the framework of the Epicost study (32) could be used to disentangle patterns of patients with homogeneous care needs and to decompose the continuing phase accordingly.

As also highlighted by Mariotto (6), since cancer incidence is highest in the elderly, the impact of population changes on cancer prevalence may exceed the impact of declining cancer incidence rates for some cancers. We are considering the possibility of incorporating the dynamics of the age structure in addition to the population changes.

For the purposes of our method, it would be worthwhile to project the initial phase prevalence stratified by stage at diagnosis. To implement this methodological enhancement, we need to retrieve information on the stage at diagnosis for initial phase patients in the last five years used as the basis for the projections.

## 5 Conclusions

Complete cancer prevalence is a fundamental but crude indicator of health service needs, as it covers all steps of the clinical pathway and includes patients with a wide range of health service requirements. Here, we presented a method, applicable where cancer registry data are available, to monitor the size of the cancer burden in a given population to define care requirements concerning the prevalence breakdown across the three phases of care, to establish priorities, and to project, in combination with average individual cost profiles, expenditures directly related to cancer care (20). For these purposes, 7 to 10 years is the time span usually considered by policymakers, and the focus is on the most frequent cancer sites that have a major economic impact on the healthcare system. Evidence from this methodology will be useful in facing the challenge of planning and developing a healthcare system that is able to respond in the short- to medium term to the increasing needs of people living with cancer.

## Data availability statement

The raw data supporting the conclusions of this article will be made available by the authors, without undue reservation.

## Author contributions

SF, AG, and SG designed the study, drafted the study protocol, collected the data, and prepared the cleaned data for the study database. FT and SG performed the statistical analyses. SF, FT, LDM, AG, and SG contributed to the validation of the statistical models and revised the statistical analyses. SF, LDM, AG, and SG discussed modeling assumptions and applicability. All authors contributed to the interpretation of the study results and reviewed and approved the final version.
