# Supplementary material for: Projecting cancer prevalence by phase of care: a methodological approach for health service planning
Source: Front Oncol. 2023 Aug 30;13:1201464. doi: 10.3389/fonc.2023.1201464 (PMC10499514; doi:10.3389/fonc.2023.1201464)

Supplementary Table 1

It compares the number of complete prevalent cases at 1st January 2018 derived by applying the projection method (PROJ) with the number of complete prevalent cases at 1st January 2018 estimated from COMPREV (ESTIMATE) by cancer site, sex and phase of care.
In general, the projection method produces slightly higher values of complete prevalence with respect to the estimation from COMPREV for colon and rectum and for lung among men, vice-versa for breast and lung cancer among women.
The two series of prevalent cases are very similar, the percent variation spanning from 3% to 10%, the maximum difference appears for colorectal cancer among men (10%), followed by colorectal and lung cancers among women (-6% and +6% respectively), and lung cancer among men and breast cancer. (+3% and -3% respectively). As illustrated in the Results, for colorectal cancer the reduction in the risk of developing the disease leading in 2007-2008 to a decrease in incidence for both men and women, does not yet compensate the combined effect of ageing and increasing survival, thus yielding a projected prevalence higher than the estimated one.

Supplementary Table 1: Complete prevalence (counts) at 1st January 2018 in the Veneto Cancer Registry area derived by applying the projection method (PROJ) and estimated from COMPREV (ESTIMATE) by cancer site, sex and phase of care. Percent variation between ESTIMATE and PROJ (VAR%) by cancer site and sex for all phases combined (total values).

	INITIAL PHASE		CONTINUING PHASE		FINAL PHASE		TOTAL		VAR%
	PROJ	ESTIMATE	PROJ	ESTIMATE	PROJ	ESTIMATE	PROJ	ESTIMATE	EST-PROJ
COLON AND RECTUM MEN	863	587	8004	7242	511	579	9378	8408	-10%
COLON AND RECTUM WOMEN	714	514	6993	6790	461	386	8168	7690	-6%
LUNG MEN	280	244	1381	1377	514	484	2175	2105	-3%
LUNG WOMEN	167	189	839	961	249	178	1255	1328	6%
BREAST WOMEN	2096	2028	28792	30190	1377	978	32265	33196	3%

Supplementary Figure 1

It compares the breakdown into the three phases of care of the complete prevalence at 1st January 2018 derived by applying the projection method (PROJ) and estimated from COMPREV (ESTIMATE) by cancer site and sex.

The two distributions appear very similar, also for those combinations of cancer site and sex with maximum percent variation between estimated and projected values, as is the case for colorectal cancer among men (see Table 1). The only striking difference between PROJ/COMPREV estimates by phase of care affects the relative proportions of the continuing and end-of-life phases for female lung cancer, which is characterized by both an increasing incidence and a poor prognosis.

Supplementary Figure 1: Percent distribution by phase of care of the complete prevalence at 1st January 2018 derived by applying the projection method (PROJ) and estimated from COMPREV (ESTIMATE) by cancer site and sex (M for men, F for women).

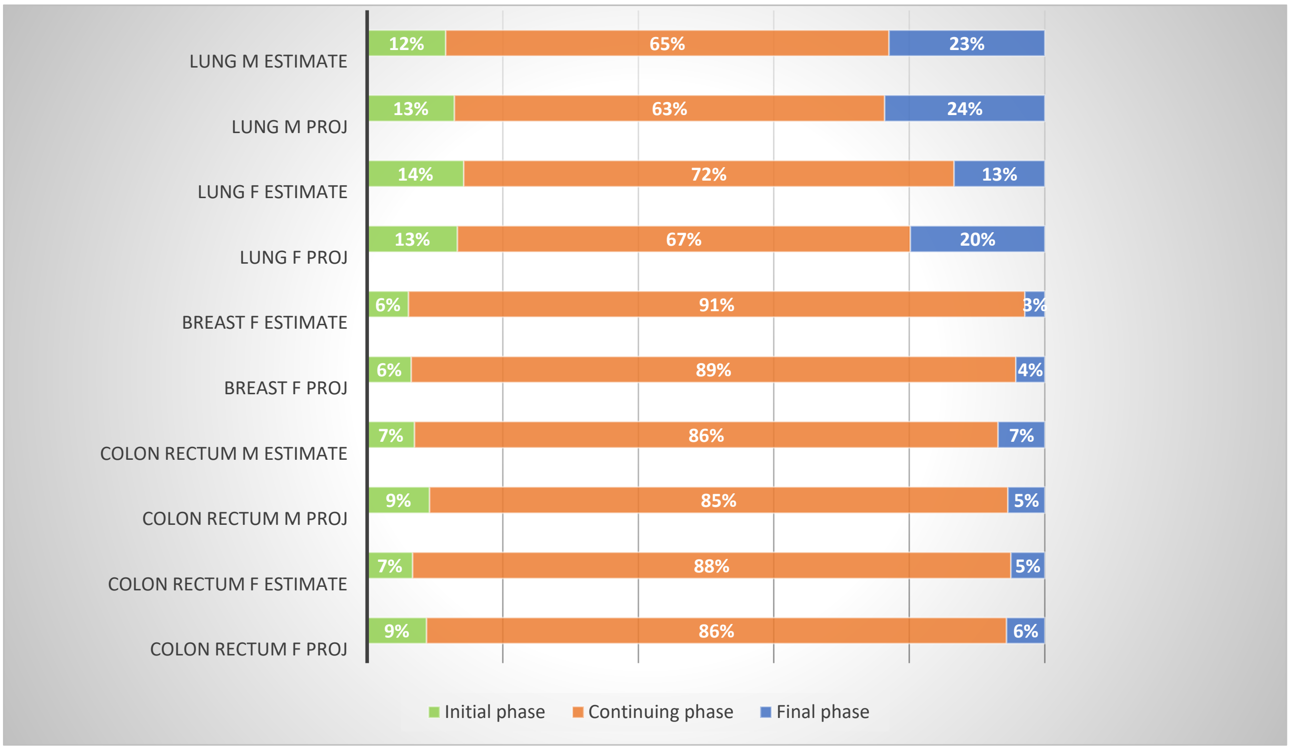

Supplementary Figure 2

It compares the complete prevalence projections in 2025 obtained using 25-year LDP data (incidence data period 1990-2018, follow-up 1st January 2019) with that obtained using 15-year LDP data (incidence data period 2000-2018, follow-up 1st January 2019), for colorectal cancer, men and women combined. In both cases, the five LDP matrices used as base for linear regression refer to the years 2015-2019, and the complete prevalence projection by phase of care refers to the year 2025.
Patterns of LDP trends (expressed as proportions per 100,000) in Supplementary Figure 2 are very similar: as expected LDP proportions obtained using 25 years of incidence data (LDP_25 and PROJ_LDP_25) are higher than those obtained using 15 years of incidence data (LDP_15 and PROJ_LDP_15), with an average percent variation of 19% during the period 2015-2026. This difference reduces to 4% when comparing the complete prevalence projections for the year 2025 obtained from 15 years data (PROJ_CP15) with complete prevalence projections for the year 2025 obtained from 25 years data (PROJ_CP25).
This result confirms the validity of the methodology proposed when CRS data availability is limited to 15 years.
The distribution of the projected complete prevalence by phase of care in 2025, is not affected by the length of incidence data series used to derive LDP (data not shown).

Supplementary Figure 2: Time trends of LDP proportions (per 100,000) for colorectal cancer (men and women combined) in the Veneto Region obtained using 25-year LDP and 15-year LDP respectively: from 2015 to 2019 proportions are based on VCR data (LDP_15, LDP_25); from 2020 to 2026 proportions are projected via linear regression (PROJ_LDP_15, PROJ_LDP_25). Complete prevalence projections in 2025 obtained from the two data series are also presented (PROJ_CP15, PROJ_CP25).

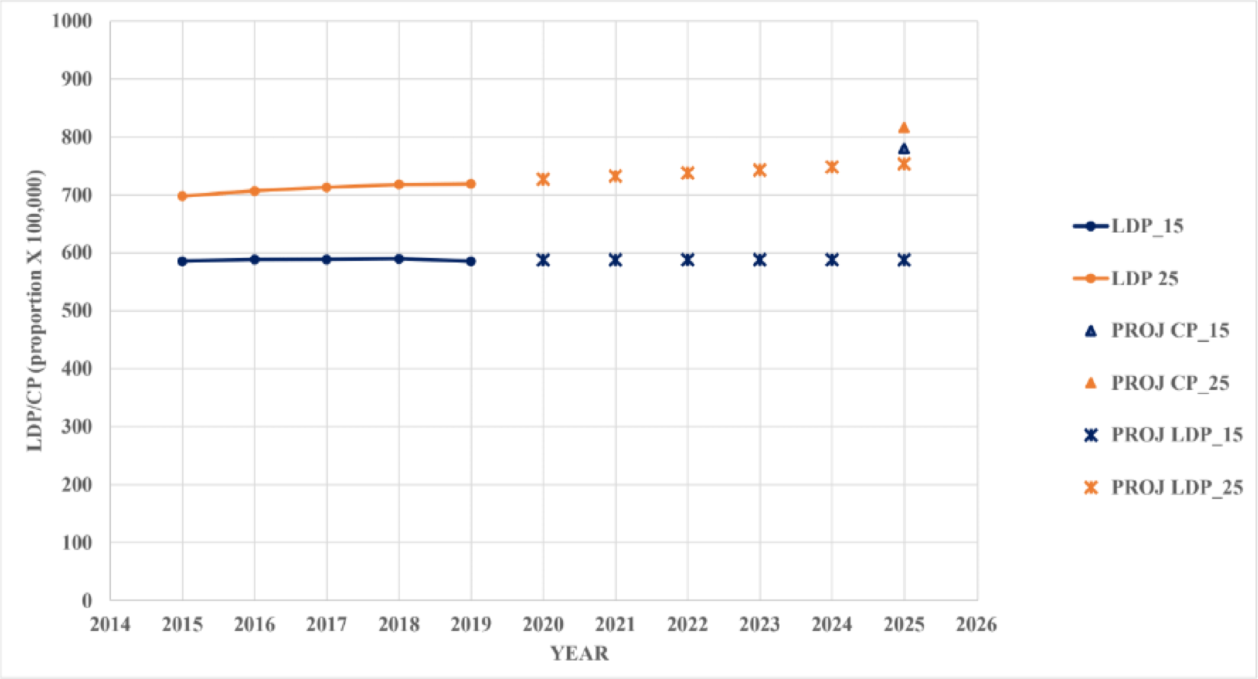

Supplement: Supplementary file 1 [file DataSheet_1.pdf]
